# Multiplex Microsphere PCR (mmPCR) Allows Simultaneous Gram Typing, Detection of Fungal DNA, and Antibiotic Resistance Genes

**DOI:** 10.1093/labmed/lmac023

**Published:** 2022-04-23

**Authors:** Daniel J Browne, Fang Liang, Kate H Gartlan, Patrick N A Harris, Geoffrey R Hill, Simon R Corrie, Kate A Markey

**Affiliations:** Division of Immunology, QIMR Berghofer Medical Research Institute, Brisbane, Australia; Centre for Molecular Therapeutics, Australian Institute of Tropical Health and Medicine, James Cook University, Cairns,Australia; Division of Immunology, QIMR Berghofer Medical Research Institute, Brisbane, Australia; Division of Immunology, QIMR Berghofer Medical Research Institute, Brisbane, Australia; School of Medicine, University of Queensland, Brisbane, Australia; Faculty of Medicine, UQ Centre for Clinical Research, University of Queensland, Royal Brisbane and Women’s Hospital, Brisbane, Australia; Division of Immunology, QIMR Berghofer Medical Research Institute, Brisbane, Australia; Division of Hematopoietic Transplantation, Fred Hutchinson Cancer Research Center, Seattle, WA, USA; Department of Chemical Engineering, ARC Centre of Excellence in Convergent Bio-Nano Science and Technology, Monash and QLD Nodes, Monash University, Clayton, Australia; Division of Immunology, QIMR Berghofer Medical Research Institute, Brisbane, Australia; School of Medicine, University of Queensland, Brisbane, Australia; Division of Hematopoietic Transplantation, Fred Hutchinson Cancer Research Center, Seattle, WA, USA; Memorial Sloan-Kettering Cancer Center, New York, NY, USA

**Keywords:** multiplex PCR, flow cytometry, MagPlex-TAG microspheres, antibiotic resistance testing, Gram typing, fungi, analytical specificity

## Abstract

**Objective:**

To show the high analytical specificity of our multiplex microsphere polymerase chain reaction (mmPCR) method, which offers the simultaneous detection of both general (eg, Gram type) and specific (eg, *Pseudomonas* species) clinically relevant genetic targets in a single modular multiplex reaction.

**Materials and Methods:**

Isolated gDNA of 16S/rRNA Sanger-sequenced and Basic Local Alignment Tool–identified bacterial and fungal isolates were selectively amplified in a custom 10-plex Luminex MagPlex-TAG microsphere-based mmPCR assay. The signal/noise ratio for each reaction was calculated from flow cytometry standard data collected on a BD LSR Fortessa II flow cytometer. Data were normalized to the no-template negative control and the signal maximum. The analytical specificity of the assay was compared to single-plex SYBR chemistry quantitative PCR.

**Results:**

Both general and specific primer sets were functional in the 10-plex mmPCR. The general Gram typing and pan-fungal primers correctly identified all bacterial and fungal isolates, respectively. The species-specific and antibiotic resistance–specific primers correctly identified the species- and resistance-carrying isolates, respectively. Low-level cross-reactive signals were present in some reactions with high signal/noise primer ratios.

**Conclusion:**

We found that mmPCR can simultaneously detect specific and general clinically relevant genetic targets in multiplex. These results serve as a proof-of-concept advance that highlights the potential of high multiplex mmPCR diagnostics in clinical practice. Further development of specimen-specific DNA extraction techniques is required for sensitivity testing.

Accurate and timely diagnosis of pathogenic microorganisms can be challenging because a wide variety of pathogens can cause clinically indistinguishable pathologies.^1^ Polymerase chain reaction (PCR)-based nucleic acid amplification tests (NAATs) offer rapid, minimally invasive, sensitive, and specific molecular diagnostics for infectious microorganisms.^2^ However, these techniques can be limited by relatively long turnaround times, a reliance upon organism culture, predefined organism panels, a lack of parallel antimicrobial susceptibility testing, and limited capacity for modular addition of further genes of interest (GOI).^1,3,4^ The ideal NAAT for use in clinical practice will have a high-multiplex capacity to identify established specific genotypes yet remain amenable to the incorporation of emerging resistance or species-specific genes in a flexible, modular fashion.

Multiplex microsphere PCR (mmPCR) is a technique that uses Cy3-labeled oligonucleotides as fluorescent reporters of primer consumption, which in turn allows the quantification of the number of copies of a given template in the specimen. When bound to carboxylated polystyrene Luminex MagPlex-TAG microspheres that are dyed into spectrally distinct sets, fluorescence intensity can be individually quantified using flow cytometry.^5^ Studies have shown that mmPCR advantageously allows high multiplex capacity (ie, theoretically capable of detecting up to 150 separate GOI in a single reaction) while maintaining the high specificity and sensitivity of PCR-based NAATs.^6^ A previously published duplex-mmPCR assay has recently been developed for rapid (ie, <3 hours), culture-free, bacterial Gram typing.^7^ In this study, we provide a significant extension to the functionality of this assay by broadening the polymicrobial detection capacity to include pan-fungal primers, specific primers targeting resistance-conferring GOIs, and species-specific primers (Supplemental Graphic Abstract). We show the high analytical specificity of our 10-plex mmPCR assay, which can simultaneously provide diagnostic information regarding Gram type, resistance genes, and specific clinically relevant pathogens.^8^

## Materials and Methods

### Oligonucleotide Design

Previously published primers were used to distinguish Gram type, fungi, β-lactamase resistance, and the specific species *Achromobacter xylosoxidans, Burkholderia cepacia,* and *Pseudomonas aeruginosa* (Supplemental Table 1). Primers targeting vancomycin type A, vancomycin type B, and methicillin resistance were designed to target the *Tn1546* genetic element, *vanB* mobile cluster, and *mecA* gene, respectively, utilizing the Primer3 software package as previously published.^9^ To facilitate mmPCR, additional nucleotides were incorporated as previously described,^5^ with MagPlex-TAG microspheres (Luminex) conjugated to DNA tags. Primers and labeled oligonucleotides were supplied as high-performance liquid chromatography grade (Integrated DNA Technologies).

### Bacterial and Fungal Genomic DNA

**Figure 1.**
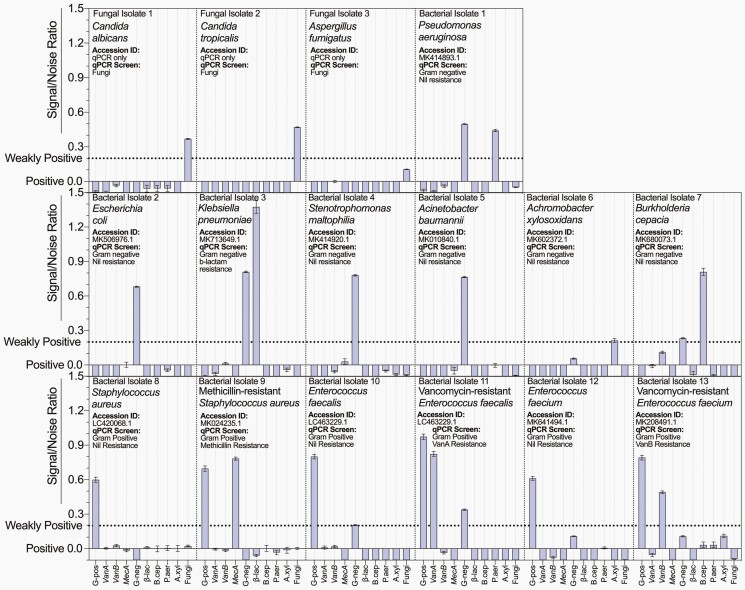
Simultaneous detection of Gram type, fungi, antibiotic resistance genes, and species-specific genes with multiplex microsphere polymerase chain reaction (mmPCR). The signal-to-noise ratio (S/N) of (13×) 16S/rRNA Sanger-sequenced and Basic Local Alignment Tool-identified bacterial isolates and (3×) fungal isolates, selectively amplified in a custom 10-plex mmPCR assay. Each 10-plex mmPCR contained general Gram-positive (G-pos), Gram-negative (G-neg) and pan-fungal (Fungi) primers; specific primers targeting the resistance-conferring gene *mecA* (MecA), the type A vancomycin resistance–conferring *Tn1546 Transposon* (VanA), the *vanB* mobile cluster (VanB), and the β-lactamases expressing *bla*_*SHV-1*_ gene (β-lac); and specific primers targeting the bacterial species *B cepacia* (B.cep), *P aeruginosa* (P.aer), and *A xylosoxidans* (A.xyl). The polymerase chain reaction was conducted using purified isolate genomic DNA at a concentration of 10^5^ genomes/reaction. The S/N of each primer set for each isolate was calculated from data normalized to a no-template negative control reaction (noise) and a “signal maximum” reaction that contained no template or forward and reverse primers (signal). An S/N >0.0 was considered positive and weakly positive when between 0.0 and 0.2 (dotted line). An S/N less than –0.1 was omitted. Data are representative of triplicate independent technical replicates. The mean ± standard error of the mean of technical triplicates is shown.

Thirteen bacterial and 3 fungal strains with characterized resistance phenotypes (Pathology Queensland) were provided from The University of Queensland and Pathology Queensland (**Figure 1**). Genomic DNA (gDNA) was extracted with a Prepito-D and Blood-600 extraction kit (Chemagen), from cultured viable cells homogenized with a Precellys 24-tissue homogenizer (Bertin Instruments) in L-type pathogen lysis tubes (Qiagen). The concentration of gDNA (genome copies/μL) was quantified with a Nanodrop ND-2000 spectrophotometer (Thermo Fisher Scientific). The identity of bacterial isolates was confirmed with Sanger sequencing (Australian Genomics Research Facility) and Basic Local Alignment Tool identification of PCR amplicons of the intervening variable 16S/rRNA regions, as previously published.^10^ The presence of antibiotic resistance genes, Gram status, and fungal presence was confirmed via real-time quantitative PCR (qPCR) as previously described.^7^ Briefly, SYBR Green (Applied Biosystems) qPCR was performed using a ViiA 7 quantitative thermocycler (Applied Biosystems), using standard cycling conditions (ie, 95°C for 10 minutes, followed by 40 cycles each of 95°C for 15 seconds and 60°C for 60 seconds, followed by a standard melt curve) with primers at 100 nmol/L. The template for amplification was 1 µL gDNA (10^5^ genome copies/μL) or no-template negative control (NTC) RT-PCR Grade Water (Life Technologies). A cycle threshold (Ct) of 35 to 30 was considered weakly positive, and a Ct <30 was considered positive. In addition, for qPCR analytical specificity testing, we tested Ct <40 as positive. All reactions were followed by a melt curve specificity test. Data were collected using QuantStudio V1.1 (Thermo Fisher Scientific) software.

### Microsphere Assay

The mmPCR assay was performed using a 2-step process as previously published,^7^ with the addition of (8×) additional primers (Supplemental Table 1). Briefly, the TaqMan Universal Master Mix (Applied Biosystems) and all forward and reverse primers were employed to selectively amplify gDNA under standard conditions (50°C for 2 minutes, 95°C for 10 minutes, followed by 45 cycles of 95°C for 30 seconds, 60°C for 30 seconds, and 72°C for 60 seconds), with a final extension step at 72°C for 5 minutes on a T100 thermocycler (BioRad). The Gram-negative, fungi, and β-lactamase primers were at 80 nmol/L; the Gram-positive, species-specific, and vancomycin B and *mecA* resistance primers were at 40 nmol/L; and the vancomycin A resistance primers were at a 20 nmol/L concentration, respectively (Supplemental Table 1). An additional 4 mmol/L MgCl_2_+ and 275 μmol/L deoxyribonucleotide triphosphate mix (Life Technologies) was added to the PCR, and the template for amplification was 1 µL gDNA (10^5^ genome copies/μL) or NTC RT-PCR Grade Water (Life Technologies). After PCR, a second hybridization stage was performed. Microspheres at a ratio of 62.5 microspheres to 1 nmol/L primer pair and Cy3-labeled oligonucleotides at a ratio of 1 nmol/L to 1 nmol/L primer pair were added and then incubated at 37°C for 30 minutes. Microspheres were analyzed on a BD LSR Fortessa II (BD Biosciences) using BD FACSdiva Software (version 8.0.1, BD Biosciences).^11^

### Data Analysis

The raw data from the BD FACSDiva Software was analyzed as previously published.^7^ Briefly, the signal/noise ratio (S/N) was calculated from data normalized to an NTC reaction (noise) and a “signal maximum” reaction that contained no template or forward and reverse primers (signal). A custom R Studio software package provided summary statistics directly from flow cytometry standard files via the bootstrap technique. An S/N >0.0 was considered positive and weakly positive when S/N values were between 0.0 and 0.2. An S/N less than –0.1 was omitted. For evaluating analytical specificity, the accuracy (%) was calculated by dividing the sum of the true positives and true negatives by the sum of all true and false positive and negatives. Positive predictive values (PPVs) and negative predictive values (NPVs) were determined using a chi-square test, and differences of accuracy between groups were assessed with a 1-way analysis of variance with Bonferroni-corrected multiple comparisons testing. In all cases, *P* < .05 was considered significant. Data are representative of triplicate independent technical replicates. Figures were prepared using GraphPad Prism version 8.4.2 (GraphPad Software).

## Results

We first sequenced each bacterial isolate included in our study to confirm that the genotype was as expected (National Center for Biotechnology Information accession number [Accession ID]; **Figure 1**). We next tested the specificity of the selected primers (Supplemental Table 1) with single-plex SYBR chemistry qPCR. To confirm the specificity of the primers, we assessed the amplification Ct for each isolate. We confirmed that the Gram-positive, Gram-negative, and fungal primers correctly typed each isolate (Supplemental Figure 1A). The *P aeruginosa, B cepacia,* and *A xylosoxidans* isolates were only amplified weakly (Ct >30) using the Gram-negative primers. Therefore, we chose these species to show the modularity of mmPCR by incorporating these species-specific primers into the assay. When we tested them using single-plex qPCR, we found that the selected species-specific primers were specific for their target isolate (Supplemental Figure 1B). In addition, we observed that the resistance gene-specific primers correctly identified the expected resistance gene expression, based on laboratory-confirmed resistance phenotype (Supplemental Figure 1C). Taken together, these data indicate that the primers selected for this study are specific when tested using single-plex qPCR.

We next sought to show that the analytical specificity of these primers is maintained when incorporated into a combined 10-plex mmPCR assay and thus show that mmPCR allows the simultaneous detection of both general and specific clinically relevant genetic targets. Using our 10-plex mmPCR, we could correctly type and identify all bacterial and fungal isolates and their resistance genes where present (**Figure 1**). Consistent with previously published data,^7^ low cross-reactive signals appeared alongside high S/N primer values. When considering the analytical sensitivity of the 10-plex mmPCR assay exclusive of weakly positive results, we found no statistically significant difference between the accuracy of qPCR and mmPCR (*P* > .9999; qPCR [Ct <30] vs mmPCR [S/N >0.2]; **Table 1**). False-positive measurements increased in frequency for both mmPCR and qPCR as the threshold of positivity was lowered (NPV = 0.9854 vs 0.8321 and NPV = 1.000 vs 0.6449 for mmPCR [S/N >0.2 vs >0.0] and qPCR [Ct <30 vs Ct <40], respectively; **Table 1**). Taken together, these data indicate that mmPCR can simultaneously detect a number of clinically relevant genetic targets and that our 10-plex mmPCR has a similar analytical specificity to single-plex SYBR chemistry qPCR.

**Table 1.** Analytical Sensitivity of mmPCR and qPCR

		Analytical Specificity				Analytical Specificity					
		Exclusive of Weakly Positive Results				Inclusive of Weakly Positive Results					
		qPCR Ct <30		mmPCR S/N >0.2		qPCR Ct <35		mmPCR S/N >0.0		qPCR Ct <40	
Pathogen or Target		TP (TN) FP (FN)	Acc (%)	TP (TN) FP (FN)	Acc (%)	TP (TN) FP (FN)	Acc (%)	TP (TN) FP (FN)	Acc (%)	TP (TN) FP (FN)	Acc (%)
General primers											
Gram-positive		6 (10) 0 (0)	100	6 (10) 0 (0)	100	6 (9) 1 (0)	93.8	6 (10) 0 (0)	100	6 (6) 4 (0)	75.0
Gram-negative		4 (9) 3 (3)	81.3	6 (7) 2 (1)	81.3	7 (9) 0 (0)	100	7 (5) 4 (0)	75.0	7 (6) 3 (0)	81.3
Fungi		3 (13) 0 (0)	100	2 (13) 0 (1)	93.8	3 (11) 2 (0)	100	3 (11) 2 (0)	87.5	3 (13) 0 (0)	100
Species-specific primers											
* B cepacia*		1 (9) 0 (0)	100	1 (15) 0 (0)	100	1 (9) 0 (0)	100	1 (13) 2 (0)	87.5	1 (1) 8 (0)	20.0
* P aeruginosa*		1 (9) 0 (0)	100	1 (15) 0 (0)	100	1 (0) 0 (0)	100	1 (12) 3 (0)	81.3	1 (2) 7 (0)	30.0
* A xylosoxidans*		1 (9) 0 (0)	100	1 (15) 0 (0)	100	1 (7) 2 (0)	80.0	1 (13) 2 (0)	87.5	1 (1) 8 (0)	20.0
Resistance-specific primers											
VanA		1 (12) 0 (0)	100	1 (15) 0 (0)	100	1 (12) 0 (0)	100	1 (12) 3 (0)	81.3	1 (12) 0 (0)	100
VanB		1 (12) 0 (0)	100	1 (15) 0 (0)	100	1 (12) 0 (0)	100	1 (11) 4 (0)	75.0	1 (6) 6 (0)	53.8
MecA		1 (12) 0 (0)	100	1 (15) 0 (0)	100	1 (12) 0 (0)	100	1 (13) 2 (0)	87.5	1 (11) 1 (0)	92.3
SHV		1(12) 0(0)	100	1 (15) 0 (0)	100	1 (12) 0 (0)	100	1 (14) 1 (0)	93.8	1 (11) 1 (0)	92.3
Chi-square test	PPV	0.8696		0.9130		1.000		1.000		1.000	
	NPV	0.9727		0.9854		0.9720		0.8321		0.6449	
1-way ANOVA			Dunn’s multiple comparisons testing					Adjusted *P* value			
			qPCR (Ct <30) vs mmPCR (S/N >0.2)					*P* > .9999			
			qPCR (Ct <30) vs qPCR (Ct <35)					*P >* .9999			
			qPCR (Ct <30) vs mmPCR (S/N >0.0)					*P* = .0233			
			qPCR (Ct <30) vs qPCR (Ct <40)					*P* = .0150			

Acc, accuracy quantified as (TP + TN)/(TP + TN + FP + FN); ANOVA, analysis of variance; Ct, cycle threshold (qPCR); FN, false negative; FP, false positive, MecA, methicillin resistance; mmPCR, multiplex microsphere polymerase chain reaction; NPV, negative predictive value; PPV, positive predictive value; qPCR, quantitative polymerase chain reaction; SHV, β-lactamase resistance; S/N, signal-to-noise ratio (mmPCR); TN, true negative; TP, true positive; VanA, vancomycin resistance type A; VanB, vancomycin resistance type B.

## Discussion

Herein, we present an mmPCR assay that can successfully detect 10 clinically relevant specific and general targets in parallel, and as proof of principle, we show its accuracy across multiple pathogens. By allowing Gram typing and establishing resistance genotypes, high-multiplex mmPCR may guide initial treatment options and minimize the use of empiric broad-spectrum antibiotics.^12^ We acknowledge that functional assays will remain critical components of resistance testing, as resistance genotype and phenotype are not always perfectly matched.^13^ However, an assessment of the likelihood of resistant organisms within ~3.5 hours is likely to be both clinically meaningful and cost-effective.^7^

By integrating species-specific primers, we have shown that this technology has the capacity for the modular addition of novel GOI targets. This capacity may facilitate the monitoring of rapidly emerging resistance or species-specific genotypes during an outbreak of a previously uncommon organism.^12^ Indeed, we suggest that the modular multiplex capacity of this mmPCR assay can be used to generate assays fit for specific patient populations in specific health care facilities, where local microbiological patterns can vary considerably. The Luminex platform allows up to 150 beads to be simultaneously detected in a single tube, although we speculate that there may be technical limitations that would prevent 150-gene detection with mmPCR. Nevertheless, our data show that high multiplexing capacity, with both specific and general primers, is possible with this platform.

This study was conducted on isolates grown from culture, with purified gDNA at a concentration of 10^5^ genome copies/reaction (ie, 0.5–0.4 ng gDNA/reaction). When considering analytical specificity, we observed low cross-reactive signals in reactions with high S/N primer values, in agreement with the previously reported Gram-typing duplex-mmPCR.^7^ Although we were able to distinguish both Gram type and resistance profile, we do accept that this finding suggests possible challenges in detecting microbial GOIs in low-biomass polymicrobial biological specimens.

When considering sensitivity, in agreement with others in the literature when testing both specific and general microbial diagnostic primers,^14^ we found that the PPV of purified and concentrated specimens was very high (0.80–1.00). We expect that this value will decrease significantly when testing clinical specimens.^15^ Indeed, the analytical and diagnostic sensitivity of a NAAT is largely dependent on the nucleic acid extraction and purification method performed, and further development of specimen-specific DNA extraction methods is required for sensitivity testing.^16^ For example, current nucleic acid isolation technologies can reproducibly isolate nucleic acids from single mammalian cells.^17^ However, no current-generation nucleic acid isolation strategy is capable of reproducibly extracting enough gDNA for analysis from the low bacterial cell numbers (ie, 0.1- to 10-colony-forming units/mL) that would be required for these methods to supersede traditional blood culture.^12^ These circumstances are despite considerable effort being devoted to the development of nucleic acid extraction technologies using combined mechanical, chemical, thermal, and enzymatic lysis strategies^18^ or increasing specimen volume.^19^

## Conclusion

We have shown a significant extension to the functionality of a previously published duplex mmPCR Gram-typing molecular diagnostic by adding clinically relevant specific and general genetic targets to generate a 10-plex mmPCR. This assay may guide treatment options via establishing Gram status, the presence of fungal DNA, and the prediction of phenotypic resistance. Furthermore, it provides a modular flexible platform that can be adapted swiftly to changes in local epidemiology.

## Supplementary Material

lmac023_suppl_Supplementary-Material

## Abbreviations:

mmPCR: multiplex microsphere polymerase chain reaction
PCR: polymerase chain reaction
NAAT: nucleic acid amplification test
GOI: genes of interest
gDNA: genomic DNA
qPCR: quantitative polymerase chain reaction
NTC: no-template negative control
Ct: cycle threshold
S/N: signal-to-noise ratio
PPV: positive predictive value
NPV: negative predictive value
