# Supplementary material for: Multiplex Microsphere PCR (mmPCR) Allows Simultaneous Gram Typing, Detection of Fungal DNA, and Antibiotic Resistance Genes
Source: Lab Med. 2022 Apr 23;53(5):459–64. doi: 10.1093/labmed/lmac023 (PMC9435484; doi:10.1093/labmed/lmac023)
Supplement: lmac023_suppl_Supplementary-Material [file lmac023_suppl_supplementary-material.docx]

| **Supplementary Table 1.** Microsphere Mediated PCR Primers and Functional Oligonucleotides | | | | | |
| --- | --- | --- | --- | --- | --- |
| **Name** | **Sequence (5’-3’)** | **Target Genetic Element** | | **mmPCR primer Concentration (nM)** | **Application** |
| **Gram-Positive Bacteria** | | | | | |
| *seq_m_*-FP | TGTATATGTTATTGAGATGTTGTAGCGRCTCTCTGGTCTGTA | | *16s* gene | 40 | Forward primer^(1)^ |
| RP | GACGACAGCCATGCASCACCTGT | |  |  | Reverse primer |
| Cy3- *seq_m_*’ | Cy3-TACAACATCTCAATAACATATACA | |  |  | Cy3-complement to *seq_m_* |
| *seq^*^* | NH_2_-TGTATATGTTA**A**TGAGATGTTGTA | |  |  | Bead tag sequence (TM37) |
| **Gram-Negative Bacteria** | | | | | |
| *seq_m_*-FP | TATGAATGTTATAGTGTGTTGATTTGAGGKATGCTGGAGGTATC | | *23s* gene | 80 | Forward primer^(2)^ |
| RP | CGACTCACCCTGCCCCGATTA | |  |  | Reverse primer |
| Cy3- *seq_m_*’ | Cy3-AATCAACACACTATAACATTCATA | |  |  | Cy3-complement to *seq_m_* |
| *seq^*^* | NH_2_-TATGAATGTTAT**T**GTGTGTTGATT | |  |  | Bead tag sequence (TM48) |
| **Pan-Fungal** | | | | | |
| *seq_m_*-FP | GTTGTAAATTGTTGTAAAGAAGTAGTGAATCATCGAATCTTTGAA | | ITS2 region | 80 | Forward primer^(3, 4)^ |
| RP | TCCTCCGCTTATTGATATGC | |  |  | Reverse primer |
| Cy3- *seq_m_*’ | Cy3-TACTTCTTTACAACAATTTACAAC | |  |  | Cy3-complement to *seq_m_* |
| *seq^*^* | NH_2_-GTTGTAAATTGT**A**GTAAAGAAGTA | |  |  | Bead tag sequence (TM15) |
| **Vancomycin Resistance - Type VanA** | | | | | |
| *seq_m_*-FP | AGTGAATGTAAGTTTATGTATTTGGGCTAGACCTCTACAGCCGA | | Tn1546 Transposon pIP816 Plasmid | 20 | Forward primer (This Study)^(5)^ |
| RP | GAGCCGGAAAAAGGCTCTGA | |  |  | Reverse primer |
| Cy3- *seq_m_*’ | Cy3-CAAATACATAAACTTACATTCACT | |  |  | Cy3-complement to *seq_m_* |
| *seq^*^* | NH_2_-AGTGAATGTAAG**A**TTATGTATTTG | |  |  | Bead tag sequence (TM13) |
| **Vancomycin Resistance - Type VanB** | | | | | |
| *seq_m_*-FP | AATGTAAAGTTAAGAAAGTGATGATTTGATCCACTTCGCCGACA | | *vanB* cluster | 40 | Forward primer (This Study) ^(6)^ |
| RP | GGGAGGATGGTGCGATACAG | |  |  | Reverse primer |
| Cy3- *seq_m_*’ | Cy3-TCATCACTTTCTTAACTTTACATT | |  |  | Cy3-complement to *seq_m_* |
| *seq^*^* | NH_2_-AATGTAAAGT**A**AAGAAAGTGATGA | |  |  | Bead tag sequence (TM44) |
| **Methicillin Resistance (mecA)** | | | | | |
| *seq_m_*-FP | TGAAATGTGTATATGTATGTTTAGACGGTAACATTGATCGCAACG | | *mecA* gene | 40 | Forward primer (This Study)^(7)^ |
| RP | TGGTCTTTCTGCATTCCTGG | |  |  | Reverse primer |
| Cy3- *seq_m_*’ | Cy3-CTAAACATACATATACACATTTCA | |  |  | Cy3-complement to *seq_m_* |
| *seq^*^* | NH_2_-TGAAATGTGTAT**T**TGTATGTTTAG | |  |  | Bead tag sequence (TM62) |
| **β-lactamase Resistance** | | | | | |
| *seq_m_*-FP | GTTGAGAATTAGTATTTGATAAAGGGGAAACGGAACTGAATGAG | | *bla_SHV-1_* gene | 80 | Forward primer ^(8)^ |
| RP | ATCGTCCACCATCCACTGCA | |  |  | Reverse primer |
| Cy3- *seq_m_*’ | Cy3-CTTTATCAAATACTAATTCTCAAC | |  |  | Cy3-complement to *seq_m_* |
| *seq^*^* | NH_2_-GTTGAGAATTAG**A**ATTTGATAAAG | |  |  | Bead tag sequence (TM73) |
| ***Achromobacter xylosoxidans* Specific** | | | | | |
| *seq_m_*-FP | GTAATTGAATTGTAAGATAAGTGTCGCATCCTGTTCCAGCA | | *bla(OXA-*114-like)  gene | 40 | Forward primer ^(9)^ |
| RP | GTGCCGGTCTTGCCATAC | |  |  | Reverse primer |
| Cy3- *seq_m_*’ | Cy3-ACACTTATCTTACAATTCAATTAC | |  |  | Cy3-complement to *seq_m_* |
| *seq^*^* | NH_2_-GTAATTGAATTG**A**AAGATAAGTGT | |  |  | Bead tag sequence (TM18) |
| ***Pseudomonas aeruginosa* Specific** | | | | | |
| *seq_m_*-FP | TTTGATTTAAGTGTGTTGAATGTAAGCGTTCGTCCTGCACAAGT | | *ecfX* gene | 40 | Forward primer ^(10)^ |
| RP | TCCACCATGCTCAGGGAGAT | |  |  | Reverse primer |
| Cy3- *seq_m_*’ | Cy3-TACATTCAACACACTTAAATCAAA | |  |  | Cy3-complement to *seq_m_* |
| *seq^*^* | NH_2_-TTTGATTTAAG**A**GTGTTGAATGTA | |  |  | Bead tag sequence (TM26) |
| ***Burkholderia cepacia* Complex Specific** | | | | | |
| *seq_m_*-FP | GATAAGAAAGTGTAATGTAAATTGCTGGAAGACATCGCGATC | | *gro* gene | 40 | Forward primer ^(11)^ |
| RP | CGTCGATGATCGTCGTGTT | |  |  | Reverse primer |
| Cy3- *seq_m_*’ | Cy3-CAATTTACATTACACTTTCTTATC | |  |  | Cy3-complement to *seq_m_* |
| *seq^*^* | NH_2_-GATAAGAAAGTG**A**AATGTAAATTG | |  |  | Bead tag sequence (TM51) |
| **^*^***seq* contains a single base mismatch (**Bold Red** letter) in comparison to *seq* | | | | | |

**Supplementary Figure 1.**

**
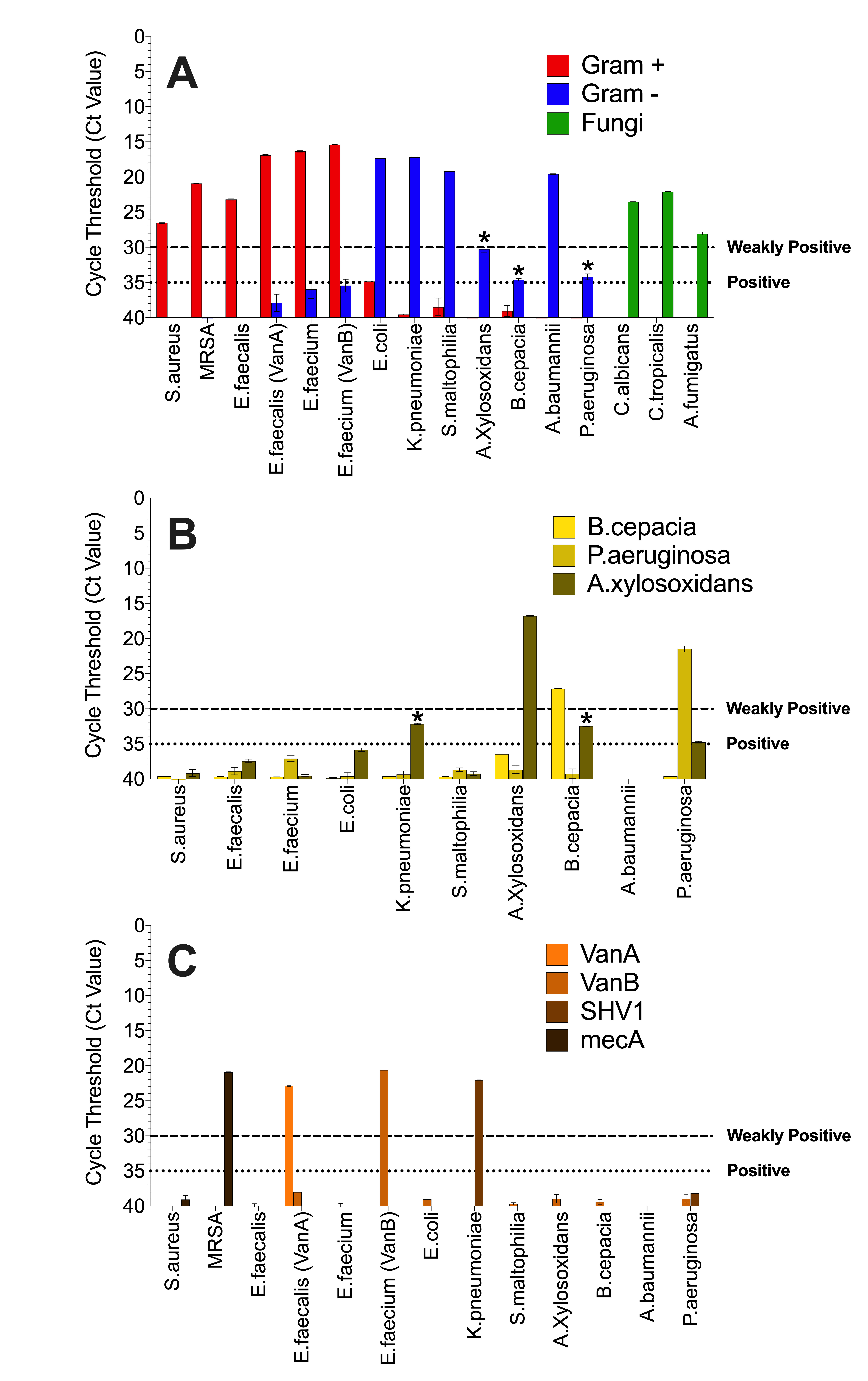
**

**Supplementary Figure 1. Testing of Gram-type, fungi, antibiotic resistance genes and species-specific genes with singleplex SYBR^®^-Chemistry quantitative PCR.** The cycle-threshold value following qPCR of bacterial (*Staphylococcus aureus,* Methicillin resistant *Staphylococcus aureus* (MRSA), *Enterococcus faecalis*, *Enterococcus faecium, Escherichia coli, Klebsiella pneumoniae, Stenotrophomonas maltophilia, Achromobacter xylosoxidans, Burkholderia cepacia, Acinetobacter baumannii* and *Pseudomonas aeruginosa*) and fungal isolates (*Candida albicans*, *Candida tropicalis* and *Aspergillus fumigatus*) when amplified in the presence of non-specific Gram-positive (red bars), Gram-negative (blue bars) and pan-fungal (green bars) primers (**A**); specific primers targeting the bacterial species *B. cepacia* (light yellow bars), *P. aeruginosa* (mid yellow bars) and *A. xylosoxidans* (dark yellow bars) (**B**); and specific primers targeting the type A vancomycin (VanA) resistance conferring *Tn1546 Transposon* (light orange bars) , the *vanB* (VanB) mobile cluster (mid orange bars), the β-lactamases expressing *bla_SHV-1_* (SHV1) gene (dark orange bars) and the *mecA* (mecA) gene (black bars) respectively. The PCR was conducted with purified isolate genomic DNA at a concentration of 10^5^ genomes/reaction. The cycle threshold value is corrected for background (amplification in no-template control). A Ct≤35 was considered positive (dotted line), while positive samples with a Ct≥30 (dashed line) were considered weakly positive (*). Data is representative of triplicate independent technical replicates. The mean ± SEM of technical triplicates are shown.

**REFERENCES**

1. Carroll NM, Jaeger EE, Choudhury S, Dunlop AA, Matheson MM, Adamson P, et al. Detection of and discrimination between gram-positive and gram-negative bacteria in intraocular samples by using nested PCR. Journal of clinical microbiology. 2000;38(5):1753-7.

2. Liang F, Browne DJ, Gray MJ, Gartlan KH, Smith DD, Barnard RT, et al. Development of a Multiplexed Microsphere PCR for Culture-Free Detection and Gram-Typing of Bacteria in Human Blood Samples. ACS Infect Dis. 2018.

3. Vancov T, Keen B. Amplification of soil fungal community DNA using the ITS86F and ITS4 primers. FEMS Microbiol Lett. 2009;296(1):91-6.

4. Turenne CY, Sanche SE, Hoban DJ, Karlowsky JA, Kabani AM. Rapid identification of fungi by using the ITS2 genetic region and an automated fluorescent capillary electrophoresis system. Journal of Clinical Microbiology. 1999;37(6):1846-51.

5. Arthur M, Molinas C, Depardieu F, Courvalin P. Characterization of Tn1546, a Tn3-related transposon conferring glycopeptide resistance by synthesis of depsipeptide peptidoglycan precursors in Enterococcus faecium BM4147. J Bacteriol. 1993;175(1):117-27.

6. Quintiliani R, Jr., Courvalin P. Conjugal transfer of the vancomycin resistance determinant vanB between enterococci involves the movement of large genetic elements from chromosome to chromosome. FEMS Microbiol Lett. 1994;119(3):359-63.

7. Malachowa N, DeLeo FR. Mobile genetic elements of Staphylococcus aureus. Cell Mol Life Sci. 2010;67(18):3057-71.

8. Hanson ND, Thomson KS, Moland ES, Sanders CC, Berthold G, Penn RG. Molecular characterization of a multiply resistant Klebsiella pneumoniae encoding ESBLs and a plasmid-mediated AmpC. J Antimicrob Chemother. 1999;44(3):377-80.

9. Turton JF, Mustafa N, Shah J, Hampton CV, Pike R, Kenna DT. Identification of Achromobacter xylosoxidans by detection of the bla(OXA-114-like) gene intrinsic in this species. Diagn Microbiol Infect Dis. 2011;70(3):408-11.

10. Clifford RJ, Milillo M, Prestwood J, Quintero R, Zurawski DV, Kwak YI, et al. Detection of bacterial 16S rRNA and identification of four clinically important bacteria by real-time PCR. PLoS One. 2012;7(11):e48558.

11. Suppiah J, Thimma JS, Cheah SH, Vadivelu J. Development and evaluation of polymerase chain reaction assay to detect Burkholderia genus and to differentiate the species in clinical specimens. FEMS Microbiol Lett. 2010;306(1):9-14.
